# The Potential Therapeutic Value of Medicinal Plants in the Management of Metabolic Disorders

**DOI:** 10.3390/molecules25112669

**Published:** 2020-06-09

**Authors:** Trevor T. Nyakudya, Thulani Tshabalala, Rachael Dangarembizi, Kennedy H. Erlwanger, Ashwell R. Ndhlala

**Affiliations:** 1 Department of Physiology, School of Medicine, Faculty of Health Sciences, University of Pretoria, Pretoria 0002, South Africa; trevor.nyakudya@up.ac.za; 2 Agricultural Research Council (ARC), Vegetable and Ornamental Plants, Private Bag X923, Pretoria 0001, South Africa; TshabalalaT1@arc.agric.za; 3 School of Agricultural, Earth and Environmental Sciences, University of KwaZulu-Natal Pietermaritzburg, Private Bag X01, Scottsville 3209, South Africa; 4 Department of Human Biology Neuroscience Institute, Faculty of Health Sciences, Division of Physiological Sciences, University of Cape Town, Anzio Road, Observatory, Cape Town 7925, South Africa; rachael.dangarembizi@uct.ac.za; 5 School of Physiology, University of the Witwatersrand, 7 York Road, Parktown 2193, South Africa; Kennedy.Erlwanger@wits.ac.za; 6 Green Technologies Research Centre of Excellence, School of Agricultural and Environmental Sciences, University of Limpopo, Private Bag X1106, Sovenga 0727, South Africa

**Keywords:** medicinal plants, metabolic syndrome, diabetes, hypertension, oxidative stress, non-alcoholic fatty liver disease

## Abstract

Metabolic syndrome (MetS) is a prevalent, multifactorial and complex disease that is associated with an increased risk of developing diabetes and other major cardiovascular complications. The rise in the global prevalence of MetS has been attributed to genetic, epigenetic, and environmental factors. The adoption of sedentary lifestyles that are characterized by low physical activity and the consumption of high-energy diets contributes to MetS development. Current management criteria for MetS risk factors involve changes in lifestyle and the use of pharmacological agents that target specific biochemical pathways involved in the metabolism of nutrients. Pharmaceutical drugs are usually expensive and are associated with several undesirable side effects. Alternative management strategies of MetS risk factors involve the use of medicinal plants that are considered to have multiple therapeutic targets and are easily accessible. Medicinal plants contain several different biologically active compounds that provide health benefits. The impact of phytochemicals present in local medicinal plants on sustainable health and well-being of individuals has been studied for many years and found to involve a plethora of complex biochemical, metabolic, and physiological mechanisms. While some of these phytochemicals are the basis of mainstream prescribed drugs (e.g., metformin, reserpine, quinine, and salicin), there is a need to identify more medicinal plants that can be used for the management of components of MetS and to describe their possible mechanisms of action. In this review, we assess the potential health benefits of South African ethnomedicinal plants in protecting against the development of health outcomes associated with MetS. We aim to provide the state of the current knowledge on the use of medicinal plants and their therapeutically important phytochemicals by discussing the current trends, with critical examples from recent primary references of how medicinal plants are being used in South African rural and urban communities.

## 1. Introduction

The prevalence of metabolic syndrome (MetS), a cluster of interrelated metabolic disorders, has been on the rise globally. The International Diabetes Federation estimated that 25% of the global adult population has MetS, and its prevalence is predicted to increase in the next few decades [1]. The rise in the prevalence of MetS poses a serious public health burden, especially in resource-limited countries in sub-Saharan Africa [2]. Several billions of dollars have already been spent by governments in developing countries to curb the widespread effects of MetS and associated risk factors [3].

The reported rise in the global prevalence of MetS and in the development of risk factors associated with it has been attributed to genetic, epigenetic, and environmental factors [4]. Also culpable in this epidemic is the adoption of sedentary lifestyles that are characterized by low physical activity or exercise and the consumption of high-energy diets [5,6]. Current management approaches for the risk factors associated with MetS involve changes in lifestyle and the use of pharmaceutical drugs that mainly target specific biochemical pathways involved in the metabolism of nutrients [7]. The pharmaceutical drugs are often expensive and characterized by poor patient compliance and have been implicated in the development of several undesirable side effects due to prolonged use. Moreover, they are monotherapeutic, usually targeting a few health outcomes associated with metabolic dysfunction. There is an urgent need to research and develop alternative and complementary approaches to the management of metabolic disorders. These alternative management strategies of MetS risk factors should involve the use of medicinal plants. Medicinal plants are defined as any plant or plant preparation(s) which provides health-promoting characteristics and temporary relief or has beneficial therapeutic and or prophylactic properties [8]. It has become acceptable to healthcare practitioners that medicinal plants have a role to play in the management and prevention of metabolic disorders [9,10]. The use of herbal medicines is not confined to developing countries but has become a multibillion-dollar industry spanning across all demographic and socio-economic strata [10].

Medicinal plants contain pharmacodynamic bioactive compounds whose additive and synergistic therapeutic effect is beneficial in the management of metabolic disorders [11,12]. Most pharmaceutical drugs are developed from medicinal plants based on local communities’ knowledge and the subsequent isolation of the main active ingredients [13]. The plant material used in preparing medicinal remedies provides potential templates for the production of pharmaceutical drugs. The discovery of beneficial phytochemical constituents in medicinal plants and their use in the management of MetS reduce the financial burden of relying on expensive synthetic pharmaceutical drugs. According to the WHO, most rural and urban-based populations in Africa still rely on traditional medicines for their primary healthcare, even in the presence of pharmaceutical drugs [14]. Another driving factor in the use of medicinal plants is the perception that they are devoid of adverse side effects and acute toxicity when compared to some of the pharmaceutical agents that are currently being used in the management of metabolic disorders [15].

Despite this perceived safety by those who prefer to use medicinal plants, there is a need for scientific validation to ensure safety and consistent medicinal preparations. In fact, the WHO is advocating for proof of safety, before establishing the therapeutic efficacy of medicinal plants to be used for primary healthcare [14]. Previously, the use of medicinal plants was based on anecdotal evidence from folklore [16]. There is indeed a plethora of information and claims about the benefits of herbal products and medicinal plants in the public press and forum. However, there is a scarcity of scientifically accurate reviews and experimental evidence on the efficacy and safety of medicinal plants. The systematic study of medicinal plants and the investigation of their biologically active phytochemical compounds for the management of metabolic disorders have emerged as a key development in modern medicine. The recent advances in molecular techniques have made it possible to intricately explore the mechanisms of action through which the phytochemical constituents of medicinal plants confer their health benefits.

We believe that the time has come to increase communication aimed at healthcare professionals and medical scientists on the advantages of using medicinal plants as safe, affordable, accessible, and natural alternative healthcare products for the management of health outcomes associated with metabolic disorders. In this review, we explore the role of medicinal plants and their phytochemicals currently being used or investigated in South Africa in treating and preventing the development of MetS risk factors such as obesity, diabetes mellitus, cardiovascular diseases, and non-alcoholic fatty liver disease.

## 2. Medicinal Plants in the Management of Obesity

There has been a worldwide recent increase in non-communicable diseases such as obesity. The WHO established that a person is overweight when his/her body mass index (BMI, calculated as kg/m^2^) is over or equal to 25 and obese when the BMI is equal to or more than 30 [17]. The global epidemic of obesity has been on the rise, mainly due to the worldwide change of diet and an increase in sedentary lifestyle [18]. It is reported that obesity cases almost tripled between 1989 and 2011 [17]. The WHO states that obesity is the seventh cause of death, and there are approximately 2.8 million people who die every year due to risk factors associated with being overweight or obese [17]. Obesity reduces life expectancy at the age of 40 by at least 7 years [19]. Recent statistics showed that in 2016, there were more than 1.9 billion adults who were overweight and 650 million people who were obese [17]. In 1998, a study indicated that about 21% of the South African population was obese [20]. Of the population, 57% of women and 29% of men were obese [21]. Furthermore, recent South African statistics show that there has been a rise of the number of obese or overweight people in the country: approximately two-thirds of South African women (68%) are obese or overweight, while 31% of men are reported to be obese [22].

Obesity occurs when there is an increase in the consumption of especially dense energy foods (carbohydrates) associated with a decrease of physical activity to burn the consumed foods [23,24]. Overweight has been associated with a myriad of comorbidities such as certain cancers (breast, prostate, kidney, colon), cardiovascular diseases (stroke and heart), and type 2 diabetes mellitus [25,26]. Recommendations to decrease or maintain weight include leading a healthy lifestyle, engaging in regular physical activity, consuming less free sugars and salts, decreasing the consumption of saturated fats and increasing that of dietary vegetables and whole grains, as well as pharmacological therapies and surgical interventions [27]. However, there is a challenge in the treatment of obesity, as only 5%–10% of individuals maintain their weight loss over the years [28]. When one ceases pharmacotherapy or abandons a healthy lifestyle, there is a reversal of the weight loss [29,30]; in addition, some of the synthetic drugs used have undesirable side effects [31,32]. Herbal supplements are some of the alternative solutions to weight loss or weight maintenance, having the advantages of being efficient, safe, and less expensive than pharmacological drugs. In this part of the review, we will list some plants that are commonly used for weight loss in South Africa, their active constituents, and their mode of action (Table 1).

## 3. South African Medicinal Plants for Diabetes Mellitus

Diabetes mellitus is one of the most common metabolic disorders in South Africa, with an incidence and prevalence that have increased at an alarming rate in the past 20 years. By 2017, the global prevalence of diabetes mellitus was estimated at 450 million [33]; about 1,826,100 people were believed to be living with diabetes in South Africa, and an additional 1,548,500 to be living with undiagnosed diabetes [33]. A large proportion of the South African population lives below the poverty datum line and has limited access to modern healthcare, thus relies on traditional medicine for managing diabetes and its complications. The subject of traditional medicines used for diabetes control is therefore a highly relevant topic in future considerations of how to deal with this condition. Through ethnobotanical studies, scientific researchers have identified a number of plant species that are used by traditional healers and herbalists in managing diabetes mellitus. In this part of the review, we will look at the use of medicinal plants in treating diabetes in South African traditional and complementary medicine.

### 3.1. Diagnosis of Diabetes Mellitus in South African Traditional Medicine

Although they have no access to laboratory-based diagnostic methods, traditional health practitioners claim they diagnose diabetes mellitus in patients using symptomatic presentations, e.g., weight loss, fatigue, excessive urination, low sex drive, and glucosuria [34,35]. The efficacy of a treatment of the condition is similarly monitored based on the reduction in symptoms, e.g., disappearance of sugar in urine, reduction in fatigue. From these reports, it is however difficult to determine if traditional diagnostic methods can distinguish between Type 1 diabetes, which is insulin-dependent, and Type 2 diabetes (not insulin-dependent) and if treatment methods are distinctive for these two. One of the challenges with an exclusively symptom-driven diagnosis is that it may be more effective for diagnosing Type 1 diabetes mellitus, in which symptoms appear early, than Type 2 diabetes mellitus, where symptoms may be less marked and delayed in their presentation, manifesting when complications have already developed.

### 3.2. Plants with Antidiabetic Activity

Extensive work has been done in South Africa in the screening of plants with antidiabetic action. Table 2 shows the list of plants identified to possess antidiabetic activity in South Africa. The list is not exhaustive, and it is possible that some of the plants used in South Africa remain unreported because of an existing gap in communication between the African traditional medicine system and modern medicine. Depending on the geographical location, different plants and/or different parts of the same plant are reportedly used for treating diabetes. Genetics and environmental factors, e.g., soil types, climate, types of vegetation, and presence of other organisms, affect the presence and levels of phytochemicals which in turn are responsible for most of the bioactivity of medicinal plants. Plants containing high levels of phenolic compounds have a high antioxidative capacity; hence, they may be useful in the treatment of diabetic complications that result from high oxidative stress [59]. Terpenes, alkaloids, and saponins may enhance insulin secretion and regulate glucose uptake and glucose utilization [60,61,62].

The modes of action of medicinal plants used in South Africa are not well reported. A few of them have been subjected to rigorous in vivo physiological, pharmacological, and biochemical testing to determine how they work in treating diabetes mellitus. *Hypoxis argentae, Tarchonanthus camphoratus, Euclea undulata, Strychnos henningsii, Cissampelo campensis, Elaeodendron transvaalense*, and *Schkuria pinnata* have been reported to increase the uptake of glucose in cultured muscle cells, hepatic cells, or preadipocytes and may therefore exhibit hypoglycemic effects through increasing the peripheral uptake of glucose [60,63,64,65]. Plants containing α-amylase and α-glucosidase inhibitors may help reduce post-prandial hyperglycemia, e.g., *Senna alexandri, Cymbopogon citrutus, Nuxia floribunda*, and *Curcubita pepo* [64]. Antidiabetic plants, e.g., *Carica papaya* and *H. argentae*, may also act by preserving and increasing the regeneration of pancreatic β-cells, hence increasing insulin release [65,66]. Some plants, e.g., *Vernonia amygdalina, Hypoxis hemerocallidea, Mimusops zeyheri, Catharanthus roseus*, and *Sutherlandia frutescens*, are very popular for the treatment of diabetes across South Africa. However, although some plants are frequently cited by traditional practitioners for their beneficial properties in the treatment of diabetes, e.g., *M. zeyheri*, there are still very few pharmacological data to validate their efficacy. Similarly, effective doses, toxicity levels, and interactions of some of these commonly used plants with modern antidiabetic pharmacological drugs remain unknown.

## 4. The Use of South African Medicinal Plants in the Management of Cardiovascular Disorders

Cardiovascular disorders (CVDs) are the leading cause of the global increase in mortality among individuals in developed [91] and, more recently, in developing countries such as South Africa [92]. CVDs and their risk factors include hypertension, myocardial infarction, angina pectoris, stroke, atherosclerosis, peripheral artery disease, and transient ischemic attack, to name but a few [93]. Most of the disease burden caused by CVDs is borne by low- and middle-income countries. CVDs are mostly prevalent in middle-aged people [94]. In the last few decades, there has been a dramatic increase in the prevalence of CVDs in South Africa [95].

Lowering high blood pressure using anti-hypertensive regimens is considered an effective way of preventing complications associated with CVDs. Most traditional anti-hypertensive pharmaceutical drugs include angiotensin receptor blockers, β-blockers, thiazide diuretics, calcium channel antagonists, and vasodilators [96]. Several plant extracts that possess therapeutic potential for the treatment of CVDs such as hypertension, atherosclerosis, ischemic heart disease, and congestive heart failure, among others, have been identified [97,98]. In the following section, we will highlight some of the important medicinal plants that are used in South Africa in the management of hypertension.

Some traditional healers have used orally administered decoctions of *Helichrysum ceres* to treat hypertension [99]. The purported hypotensive effects of this plant extract have been attributed to the presence of natriuretic and diuretic bioactive phytochemical compounds [100]. Moreover in vivo studies have also shown that the ethanolic leaf extracts of *H. ceres* act on vascular smooth muscles, causing a vasodilatory effect which in turn reduces the total peripheral resistance (TPR), thus lowering blood pressure [99]. *Ekebergia capensis* leaf ethanolic extracts have also been used successfully to prevent the development of hypertension in murine models [101]. The hypotensive effects of *E. capensis* are due to its modulatory effects on the TPR of the vascular smooth muscles [101].

*Opuntia megacantha* crude leaf extracts have been shown to reverse the inability of kidneys to excrete sodium in a streptozotocin-induced (STZ) diabetic rat model [102]. This suggests that the plant extracts may have beneficial effects in the management of hypertension through its influence on kidney’s ability to regulate the blood volume. The other South African medicinal plants that are popularly used in the management of hypertension due to their vasorelaxant, bradycardiac, and cardioprotective effects include *Allium sativum* (phenols and flavonoids) [103], *Sclerocarya birrea* (flavonoids and triterpenes) [104], *Ficus thonningii* (anthraquinones, flavonoids, and saponins) [105], and *Olea europea* (triterpenes, flavonoids, and glycosides) [106]. The phytochemicals isolated from wild African olive leaves (*Olea europea*) from Cape Town exhibited diuretic, anti-atherosclerotic, and anti-hypertensive effects [107]. Experimental animal studies using an insulin-resistant rat model showed that six-week treatments with *O. europea* extracts prevented the development of hypertension and atherosclerosis, demonstrating the potential of this medicinal plant in the management of hypertension in the African population [107].

Phytochemical constituents in anti-hypertensive medicinal plant preparations also target the renin–angiotensin–aldosterone system (RAAS), a key signaling pathway fundamental in blood pressure regulation. The angiotensin-converting enzyme (ACE) plays an important role in the development of hypertension by converting angiotensin I to angiotensin II [108,109]. The inhibition of ACE activity is evaluated when screening for anti-hypertensive medicines (Morgan et al., 2001). Several in vitro studies were done on South African medicinal plants to investigate their ACE inhibition potential. It has been shown that aqueous and ethanolic extracts of *Stangeria eriopus, Amaranthus dubius, Amaranthus ybridus, Asystasia gangetica, Galinsoga parviflora, Justicia flava, Oxygonum sinuatum, Physalis viscosa*, and *Tulbaghia violacea* exhibited ACE inhibitor activity. *T. violacea* had the highest ACE inhibitor activity [110], demonstrating its potential in the treatment of hypertension. The ACE inhibition observed in most of these medicinal plants is due to the presence of tannins which interfere with ACE activity [111]. Common plants that are used in the treatment of cardiovascular diseases are shown in Table 3.

## 5. South African Medicinal Plants for Non-alcoholic Fatty Liver Disease

Non-alcoholic fatty liver disease (NAFLD) is a major cause of morbidity and mortality worldwide. Although non-alcoholic fatty liver disease is generally associated with obesity and has been considered as the metabolic manifestation of MetS, recent evidence shows that it can develop independently of metabolic syndrome [112]. The diagnosis of NAFLD requires the use of either diagnostic biopsies or advanced technology. Consequently, traditional medical practitioners are highly unlikely to be able to diagnose NAFLD in patients. Therefore, in reporting on medicinal plants used for the management of or showing biological activity against NAFLD, this review will focus on South African plants which have been confirmed to demonstrate activity against hepatic steatosis. The available literature is also mainly from lab animal studies. High-fructose diets have been implicated in the development of NAFLD. We have recently shown that aqueous extracts of *Terminalia sericea* leaves prevented the development of NAFLD in Wistar rats fed a high-fructose diet for 12 weeks [113].

Herbal tea made from the aerial parts of Rooibos *Aspalathus linearis* has been shown to have multiple health benefits. These health benefits have been mainly attributed to the C-glucosyl dihydrochalcone aspalathin, which is especially enriched in the fermented tea. In vitro studies have shown that aspalathin and aspalathin-enriched green tea improved lipid metabolism in adipocyte-derived 3T3-L cells [114]. Further studies showed that an aspalathin-enriched green Rooibos extract was also able to inhibit hepatic insulin resistance in vitro in hepatic cells and in vivo in obese insulin-resistant rats, through mechanisms that involved the regulation of AMP-activated protein kinase (AMPK) pathways, amongst others [115].

Dietary modifications, including a restricted food intake, are recognized as a potential therapeutic approach for the management of obesity and NAFLD. The succulent plant *Hoodia gordonii* has been used by the Khoi San for decades as an appetite suppressant. Through its appetite-suppressing effects *H. gordonii* is a potential candidate for the management of NAFLD. While some studies showed varied results in humans [39], recent studies using obese rats showed that dietary supplementation with *H. gordonii* extracts decreased the rats’ body mass and reduced their muscle mass and adipocyte size [116]. There is a need to explore the use of *H. gordonii* in the management of NAFLD. The legume *S. frutescens* is widely used for its medicinal properties. As discussed earlier, recent studies have validated its antidiabetic properties, and it has also been shown to modify lipid metabolism in 3T3 adipocytes as well as in insulin-resistant rats [117]. Importantly, it has also been shown that its hot aqueous extracts were able to reverse fructose-induced hepatic steatosis in vivo [46].

*Aloe vera* is renowned for its medicinal efficacy against hepatic steatosis, and it has been demonstrated that *A. vera* gel extracts ameliorated this condition in rats. One of the major bioactive compounds in *A. vera* thought to be responsible for the hepatoprotective effects is kaempferol [118]. In addition, other phytosterols found in *A. vera* (lophenol and cycloartanol) significantly decreased the expression of lipogenic genes and reduced hepatic lipid accumulation when administered to Zucker diabetic fatty rats [42].

In a study in which we fed rats a high-fructose diet, it was shown that methanolic leaf extracts of *Moringa oleifera* were able to prevent the development of NAFLD [119]. Using extracts from fermented *M. oleifera*, hepatic lipid accumulation was decreased in high-fat diet-induced obese mice, possibly through mechanisms which involved the upregulation of genes associated with lipid hepatic metabolism and the suppression of hepatic pro-inflammatory cytokine mRNA expression [120]. *Opuntia ficus* seed extract (DWJ504) when fed to rats in the last four weeks of a 10-week high-fat diet, prevented hepatic steatosis, induced macrophage polarization, and suppressed inflammatory signaling pathways associated with Toll-like 4 receptors, tumor necrosis factor alpha, and interleukin 6 [121].

In addition to *Syzigium aromaticum* cold-pressed oil, which was reported to be hepatoprotective against chemically induced hepatotoxicity [122], oleanolic acid isolated from *S. aromaticum* was shown to induce protection against high-fructose diet-induced NAFLD when it was fed to neonatal rats during the suckling period [123]. Thus, there are several plants with demonstrated efficacy for prophylaxis and/or treatment of NAFLD in experimental models of the disease (Table 4). There is, however, a need to explore the potential of these plants and their biologically active constituents in humans.

## 6. Conclusions

Non-communicable diseases as well as the risk factors for MetS contribute significantly to the burden of health care in South Africa. There is a rich biodiversity of plants in South Africa with demonstrated and scientifically validated medicinal properties, which are and can be exploited in reducing the burden of provision of health care. The role of the gut microbiota in metabolic diseases has gained a lot of attention. Several of the plants discussed in this review contain phytochemicals which have selective microbicidal activity which can alter the Bacteroidetes-to-Firmicutes ratio, imparting positive health beneficial effects [124]. Non-digestible fibers and phytochemicals such as polyphenols present in some of the plants could also have positive prebiotic effects [125]. However, whilst traditional practitioners may target specific gastrointestinal disorders, they do not have the capacity to assess the impact of their interventions on the gut microbiota. There is a need for further studies and, indeed, for a specific review dedicated to this important subject. However, what is clear from our review is that there is a need to protect plant biodiversity and ensure that plants are not over-exploited, as has been reported in China [126]. Nevertheless, as this review has shown, the development of novel plant-derived medicines for the management of MetS has immense potential and should not be ignored in the effort to combat the impact of MetS on health care delivery.

## Figures and Tables

**Table 1:** Plants in South Africa used for weight loss.

Family Name	Species Name	Common Name	Plant Part Used	Methods of Herbal Material Preparation	Mode of Action	Active Constituents	References
Apiacaea	*Foeniculum vulgare* Mill	Fennel	Seeds	The seeds are processed into powder which can be taken as an infusion	Reduces oxidative stress, inhibits serotonin reuptake, promotes a decrease in fat and sugar absorption	Phytoestrogens, dipentene	[36]
Apocynaceae	*Gymnema sylvestre* R. Br	Gimena	Leaves	The leaves are used to make an infusion taken orally	Inhibits glucose absorption and fatty acid accumulation	Gymnemic acids	[37,38]
Apocynaceae	*Hoodia gordonii* (Masson)	Kalahari cactus	Stem	Tender stems are eaten fresh or dried and milled. Often processed into capsules	Appetite suppressant targets adipose and muscle tissues reduces calorie intake	Oxypregnane steroidal glycoside P57	[39]
Asphodelaceae	*Aloe ferox* Miller	Cape Aloe	Leaves	Leaves are taken as decoctions	Combats water retention		[40]
Asphodelaceae	*Aloe vera* Mille	Aloe vera	Leaves	Leaves are taken as decoctions	Improves carbohydrate metabolism and reduces obesity-induced glucose intolerance	Aloe sterols	[41,42]
Asteraceae	*Taraxacum officinale* F.H. Wigg.	Dandelion	Leaves	Leaves are taken as decoctions	Inhibits pancreatic lipase	Caffeic and chlorogenic acid	[43,44]
Cannabaceae.	*Cannabis sativa* L.	Marijuana	Leaves	The leaves are used to make an infusion taken orally	Psychoactive rapid and long-lasting downregulation of CB1R causes reduction of energy storage and increases metabolic rates	Cannabinoids	[45,46,47]
Cucurbitaceae	*Cucumis africanus* L.f.	Scarlet gourd	Whole plant	The plant is used to make an infusion taken orally	Weight loss	Flavonoids	[48,49]
Cucurbitaceae	*Kedrostis africana* (L.) Cogn.	Baboon′s cucumber	tuber	The tuber is used to make a decoction which is taken orally	α-amylase, α-glucosidase, and lipase inhibitory activities	Luteolin and kaempferol	[50]
Curtisiaceae	*Curtisia dentata* (Burm.f.) C.A. Sm.	Assega	Bark	The bark is used to make a decoction which is taken orally	Weight loss		[45]
Fabaceae	*Acacia mearnsii* De Wild	Black wattle	Bark	The bark is used to make a decoction which is taken orally	Increases energy expenditure in skeletal muscle and decreases fatty acid synthesis	Proanthocyanidins,	[51,52]
Lamiaceae	*Rosmarinus officinalis* L.	Rosemary	Leaves	The leaves are used to make a decoction which is taken orally	Reduces body fluid	Carnosic acid	[53]
Menispermaceae	*Cissampelos capensis* L.f.	David root	Roots	The root is used to make a decoction which is taken orally	Stimulates body energy		[45]
Moringaceae	*Moringa oleifera* Lam.	Moringa	Leaves	The leaves are used to make a decoction which is taken orally	Lowers body weight, total cholesterol, triglycerides, organ weight, and blood glucose level, promotes energy expenditure	Quercetin-3-O-β-dglucoside	[54,55,56]
Poaceae	*Coix lacryma-jobi* L.	Job’s tears	Seeds	The seeds are used to make a decoction which is taken orally	Neuroendocrine activity downregulation of adipogenesis		[57]
Polygonaceae	*Persicaria hydropiper* (L.) Spach.	Water pepper	Leaves	The leaves are used to make infusions which are taken orally	Combats adipogenesis in 3T3-L1 cells	Isoquercitrin	[58]

**Table 2:** List of plants used for the treatment of diabetes mellitus in South Africa.

Family Name	Scientific Name	Local Name and Region Where Used	Plant Part Used	Methods of Herbal Material Preparation	Mechanisms	Scientific Model Used	Reference
Aizoaceae	*Carpobrotus edulis (L.) N.E. Br*	-	Leaves	The leaves are used to make an infusion which is taken orally	-	-	[67]
Alliaceae	*Allium sativum L. fam.*	Garlic (English) Ivimbampunzi (IsiXhosa) Ikonofile (IsiZulu); Eastern Cape	Whole plants	The different parts are used to make a decoction which is taken orally	Hypoglycemic, hypolipidemic; reduces proteinuria	STZ-treated rats	[68]
Amaryllidaceae	*Gethyllis namaquensis (Schönland) Oberm.*	Naka tsa tholo; Limpopo Province	Bulbs	Aqueous extract which is taken orally	-	-	[67]
Anacampserotaceae	*Anacampseros ustulata E.Mey. ex Fenzl*	Igwele (IsiXhosa); Eastern Cape	Corms		-	-	[35]
Anacardiaceae	*Sclerocarya birrea (A. Rich) Hochst. Subsp caffra*	Cider/Marula (English) Maroela (Afrikaans) Umganu (Zulu)	Bark	The bark is used to make a decoction which is taken orally	Reduces blood glucose, increases insulin levels	STZ-treated rats	[69,70]
Apocynaceae	*Catharanthus roseus (L.) G.Don*	Madagascar periwinkle	Leaves, whole plants	The leaves are used to make an infusion which is taken orally	Increased expression of *GLUT-2* and *GLUT4* transporter gene expression in the liver Hypoglycemic; hypolipidemic; increases the activity of glycolytic pathway enzymes; activates nuclear peroxisome proliferator and hence regulates gene expression in metabolic pathways; upregulates glucokinase activity	STZ-treated rats; alloxan-treated rats; in vitro enzyme assays; alloxan-treated rabbits cultured human cells	[71,72,73,74]
Apocynaceae	*Plumeria obtusa L.*	Mohlare wa maswi wa sukiri; Limpopo Province	Leaves	The leaves are used to make an infusion which is taken orally	-	-	[67]
Araliaceae	*Cussonia spicata Thunb.*	Limpopo Province	Roots	The root bark is used to make a decoction which is taken orally	-	-	[67]
Asphodelaceae	*Aloe ferox Mill*	Ikhala (IsiXhosa) Bitter Aloe (English); Eastern Cape	Leaves	The leaves are used to make an infusion which is taken orally	Hypoglycemic; increases insulin secretion	STZ-treated rats	[40,75]
Asphodelaceae	*Aloe marlothii A. Berger subsp. Marlothii*	-			-	-	[67]
Asphodelaceae	*Bulbine abyssinica A.Rich.*		Whole plants	Different parts of the plant are used to make into a which is taken orally	-	-	[59]
Asphodelaceae	*Bulbine frutescens (L.) Willd.*	Ibhucu (IsiXhosa); Eastern Cape	Roots	The root is used to make a decoction which is taken orally	-	-	[34]
Asphodelaceae	*Bulbine natalensis (Syn. B. latifolia) Mill. (L.f.) Roem. et Schult.*	Ibhucu (IsiXhosa); Eastern Cape	Roots	The root is used to make a decoction which is taken orally	-	-	[34]
Asteraceae	*Artemisia afra Jacq. ex Willd.*	Umhlonyane (IsiXhosa) African wormwood	Leaves, roots	The roots are used to make a decoction; leaves are used to make a decoction which is taken orally	Hypoglycemic and hypolipidemic effects	STZ-treated Wistar rats	[76]
Asteraceae	*Brachylaena discolor DC.*		Leaves, roots, and stems	The roots are made into a decoction which is taken orally	Inhibits α-amylase and α-glucosidase; increases glucose utilization in Chang liver cells, 3T3-L1, and C2C12 muscle cells	In vitro enzyme assays; in vitro cultures of preadipocytes, hepatocytes, and muscle cells	[77,78]
Asteraceae	*Callilepis laureola DC.*	Phela (Sepedi); Limpopo Province	Roots	The roots are used to make a decoction which is taken orally	-	-	[67]
Asteraceae	*Helichrysum caespititium (DC) Harv.*	Bokgatha/Mabjana/Mmeetse; Limpopo Province	Whole plant	The different parts are used to make a decoction which is taken orally	-	-	[67]
Asteraceae	*Helichrysum gymnocomum DC. var. acuminatum DC.*	Imphepho (Xhosa); Eastern Cape	Leaves	The leaves are used to make an infusion which is taken orally	-	-	[67]
Asteraceae	*Herichrysum odoratissimum L.*	Imphepho; Eastern Cape	Whole plant	The different parts are used to make a decoction which is taken orally	-	-	[34]
Asteraceae	*Herichrysum nudifolium L.*	Ichocholo; Eastern Cape	Leaves, roots	The leaves are used to make an infusion which is taken orally	-	-	[34]
Asteraceae		-	-		Increases glucose uptake in Chang liver cells, 3T3-L1 pre-adipocytes	In vitro cultures of preadipocytes and hepatocytes	[60]
Asteraceae	*Tarchonanthus camphoratus L.*	Limpopo Province, Eastern Cape	Roots, leaves/soft twigs	The leaves are used to make an infusion which is taken orally	Increases glucose utilization in Chang liver cells and C2C12 muscle cells	In vitro cultures of myocytes and hepatocytes	[79]
Asteraceae	*Herichrysum petiolare H & B.L.*	Imphepho; Eastern Cape	Whole plant	The different parts are used to make a decoction which is taken orally	-	-	[34]
Buddlejaceae	*Chilianthus olearaceus Burch.*	Umgeba (IsiXhosa); Eastern	Leaves, and twigs	The twigs are used to make a decoction which is taken orally	-	-	[34]
Cactaceae Opuntia	*ficusindica Mill.*	Motloro; Limpopo Province	Roots	The roots are used to make a decoction which is taken orally	Hypoglycemic	Type 2 diabetic patients; STZ-treated mice; alloxan-treated mice	[80,81]
Caricaceae	*Carica papaya L.*	Mophopho; Limpopo Province	Leaves, toots, seeds, pulp	The leaves are used to make an infusion which is taken orally	Hypoglycemic, hypolipidemic; increases the regeneration of pancreatic β-cells and renal cuboidal cells; anti-atherogenic	STZ-treated rats; alloxan-treated rats	[66,82,83]
Caryophyllaceae	*Dianthus thunbergii S.S.Hooper forma thunbergii.*	Indlela-zimhlope	Roots	The roots are used to make a decoction which is taken orally	-	-	[35]
Celastraceae	*Elaeodendron transvaalense (Burtt Davy) R.H.Archer*	Venda, Limpopo	Stembark	The stem barks are used to make a decoction which is taken orally	Increases glucose uptake in 3T3-L1 pre-adipocytes	In vitro cultures of preadipocytes	[60]
Celastraceae	*Lauridia tetragona (L.f.) R.H.Archer*	Umdlavuza; Eastern Cape	Barks		-	-	[35]
Cucurbitaceae	*Cucurbita pepo L.*	Intsunga (pumpkin leaves) Newcastle KZN	Upper parts (leaves and stems)		α-glucosidase activity in vitro	In vitro enzyme assays	[64]
Cucurbitaceae	*Momordica balsamina L.*	Mothwatwa; Limpopo Province	Roots		-	-	[67]
Cucurbitaceae	*Mormordica charantia L*	Monamelala; Limpopo Province	Leaves, fruit	The leaves are used to make an infusion which is taken orally	Hypoglycemic, hypolipidemic	Diabetic patients; STZ-treated rodent models	[84,85]
Ebenaceae	*Euclea undulata Thunb.*	Venda, Limpopo	Rootbark	The root bark is used to make a decoction which is taken orally	Increases glucose uptake in Chang liver cells, 3T3-L1 pre-adipocytes, and C2C12 myocytes; inhibits α-glucosidase activity	In vitro cultures of preadipocytes, myocytes, and hepatocytes; in vitro enzyme assays	[60]
Fabaceae	*Lessertia microphylla (Burch. Ex DC.)* *Goldblatt & J.C. Manning*	Mosapelo; Limpopo Province	Roots	The roots are used to make a decoction which is taken orally	-	-	[67]
Fabaceae	*Senna alexandria Mill.*	Senna leaves; Newcastle KwaZulu-Natal	Leaves	The leaves are used to make an infusion which is taken orally	Inhibits α-amylase and α-glucosidase activity in vitro	In vitro enzyme assays	[64]
Fabaceae	*Sutherlandia frutescens (L.) R.Br.*		Leaves, shoots		Hypoglycemic, increases glucose uptake in muscle and adipose tissue	STZ-treated rats	[86]
Hyacinthaceae	*Albuca setosa Jacq.*	Eastern Cape	Corms				[35]
Hyacinthaceae	*Hypoxis argentae L.*		Corms		Increases glucose uptake in cultured L6 myotubes and HepG2 cells; increases pancreatic beta cell proliferation	In vitro cultures of rat skeletal muscle cells, human hepatocellular carcinoma cells, and	[65]
Hyacinthaceae	*Hypoxis colchicifolia Bak.*	Inongwe; Eastern Cape	Corms		-	-	[34]
Hyacinthaceae	*Hypoxis hemerocallidea Fisch. & C. A*	African potato Inongwe; Eastern Cape	Corms		Hypoglycemic ethyl acetate extract inhibits α-amylase and α-glucosidase activity in vitro; acetone extract increases insulin release from cultured islet cells	STZ-treated rats, in vitro enzyme assays; cultured Sprague Dawley rat pancreatic islet cells	[64,85,87,88]
Hyacinthaceae	*Hypoxis iridifolia Baker Monna maledu; Limpopo Province*	Monna maledu; Limpopo Province			-	-	[67]
Lamiaceae	*Leonotis leonorus (L.) R.Br.*	wild dagga, lion′s ear, leonotis (Eng.); wildedagga, duiwelstabak (Afr); umfincafincane, umcwili, imunyane, utshwalabezinyoni (isiZulu)	Whole plants	The different parts are used to make a decoction which is taken orally	Hypoglycemic, hypolipidemic	STZ-treated rats	[89,90]
Loganiaceae	*Strychnos henningsii Gilg*				Hypoglycemic, hypolipidemic; increases insulin sensitivity in 3T3- L1 cells	STZ-treated rats; in vitro cultures of rat pre-adipocytes	[35,61]
Menispermaceae	*Cissampelo capensis L.*	Umayisake (IsiXhosa)/David root (English); Eastern Cape	Roots	The roots are used to make a decoction which is taken orally	Improves glucose utilisation in 3T3- L1 cells	In vitro cultures of preadipocytes	[45,77]
Poaceae	*Cymbopogon citrutus Stapf*	Isiqunga (lemon grass); Newcastle KZN	Whole plant	The different parts are used to make a decoction which is taken orally	Inhibits α-amylase and α-glucosidase activity in vitro	In vitro enzyme assays	[64]
Sapotaceae	*Mimusops zeyheri Sond.*	Mmupudu; Limpopo Province			-	-	[67]
Solanaceae	*Solanum aculeastrum Dunal*	Umtuma; Eastern Cape	Roots	The roots are used to make a decoction which is taken orally			[61]
Stilbaceae	*Nuxia floribunda Benth.*	Umlulama (forest elder) Newcastle KZN	Whole plant	The different parts are used to make a decoction which is taken orally	α-glucosidase activity in vitro	In vitro enzyme assays	[64]

**Table 3:** Plants used in the management of cardiovascular diseases in South Africa.

Family Name	Scientific Name	Local Name and Region Where Used	Plant Part Used	Methods of Herbal Material Preparation	Mechanisms	Scientific Model Used	Reference
Asteraceae	*Helichrysum ceres*	Blombos straw flower (English); Izangume (Zulu) Northern Cape, Western Cape	Leaves	The leaves are used to make an infusion which is taken orally	Hypotensive	Dahl salt-sensitive genetically hypertensive rats	[99]
Meliaceae	*Ekebergia capensis*	Cape ash, dogplum (English) Essenhout (Afrikaans); Mmidibidi (Nothern Sotho). Eastern Cape, KwaZulu-Natal, Limpopo, Mpumalanga	Leaves	The leaves are used to make an infusion which is taken orally	Hypotensive	Streptozotocin- induced diabetic rats	[101]
Cactaceae	*Opuntia megacantha*	Sweet prickly-pear (English); turksvy (Afrikaans); itolofiya (Xhosa). Widely distributed in South Africa	Fruit	The leaves are used to make an infusion which is taken orally	Hypotensive	Streptozotocin- induced diabetic rats	[102]
Amaryllidaceae	*Allium sativum*	Garlic (English), Knoffelhuisies (Afrikaans). Non-indigenous	Cloves	Cold-pressed extract	Attenuation of structural nephropathy progression	Streptozotocin-induced diabetic rats	[103]
Anacardiaceae	*Sclerocarya birrea*	Marula (English); Morula (Southern Sotho). KwaZulu-Natal, Limpopo, Mpumalanga	Leaves	The leaves are used to make an infusion which is taken orally	Phenolic compounds hypotensive	*In vitro* analyses	[104]
Moraceae	*Ficus thonningii*	Giant-leaved fig (English); Reuseblaarvy (Afrikaans); Umvubu, Omkhulu (Zulu); Umthombe, uluzi (Xhosa), KwaZulu-Natal	Stem bark	Stem–bark aqueous extract	Decrease in mean arterial pressure	Anesthetized rat model	[105]
Oleaceae	*Olea europea*	Wild olive (English), Olienhout (Afrikaans); Mohlware (Nothern Sotho, Southern Sotho), Umnquma (Zulu, Xhosa, Swati). Widely distributed in South Africa	Leaves	The leaves are used to make a decoction which is taken orally	Diuretic, anti-atherosclerotic, and anti-hypertensive effects	Insulin-resistant genetic rodent models	[106,107]
Amaryllidaceae	*Tulbaghia violacea*	Wild garlic or society garlic (English), Wildeknoflok (Afrikaans); Utswelane (Xhosa); Incinsini (Zulu). Eastern Cape, KwaZulu-Natal, Limpopo	Leaves and flowers	The leaves are used to make a decoction which is taken orally	ACE inhibitors	In vitro assays	[110,111]

ACE, angiotensin-converting enzyme.

**Table 4:** List of plants with laboratory-tested potential for use in the treatment of non-alcoholic fatty liver disease in South Africa. AMPK, AMP-activated protein kinase.

Family Name	Scientific Name	Local Name and Region Where Used	Plant Part Used	Methods of Herbal Material Preparation	Mechanisms	Scientific Model Used	Reference
Moringaceae	*Moringa oleifera*	Moringa, Drum stick tree (English) Limpopo province and Gauteng	Aerial	Leaf extracts	Upregulation of hepatic lipid metabolism genes, suppression of pro-inflammatory pathways	Diet-induced obesity models in rats,	[119,120]
Fabaceae	*Aspalathus linearis* (Burm.f.) R.Dahlgren	Rooibos, red bush (English) Rooibostee, bossietee (Afrikaans) Fynbos, Northern and Western cape	Aerial parts	Aerial parts as tea, green or fermented	Improved lipid metabolism in adipocytesinhibit hepatic insulin resistanceregulation of AMPK	3T3-L adipocyte cell culture, obese insulin-resistant rats	[114,115]
Apocynaceae	*Hoodia gordonii*	Bitter ghaap (English); Muishondghaap, wolweghaap, bergghaap, bokhorings (Afrikaans); khobab (Khoi)	Aerial		Appetite suppression, decrease adipocytes	Obese rats	[116]
Fabaceae	*Sutherlandia frutescens*	Cancer bush (English), kankerbos (Afrikaans) Western cape	Leaves	Decoction, aqueous extracts	Regulation of adipocytes and lipid metabolism	3T3 cells and obese rats	[46,117]
Asphodelaceae	*Aloe vera*	Aloe (English); Aalwyn (Afrikaans); Hlaba, Lekhala (Southern Sotho); Icena (Ndebele); Imboma (Zulu). Widely distributed in South Africa	Leaves	Gel extract	Phytosterols, decrease hepatic lipid accumulation	Zucker obese rats	[42]
Cactaceae	*Opuntia ficus-indica (L) Mill*	Prickly pear (English), Limpopo province	Fruit	Seed extracts	Decrease inflammation, prevent steatosis	High-fat fed rats	[121]
Myrtaceae	*Syzigium aromaticum*	Clove (English), Naeltjies (Afrikaans). Non-indigenous, grown as ornamental tree	Cloves	Cold-pressed extract (oleanolic acid)	Developmental programming Regulation of hepatic lipid metabolism pathways	High-fructose-fed rats	[123]

## References

[1] O’neill S., O’driscoll L. (2015). Metabolic syndrome: a closer look at the growing epidemic and its associated pathologies. Obes. Rev..

[2] Nolan P.B., Carrick-Ranson G., Stinear J.W., Reading S.A., Dalleck L.C. (2017). Prevalence of metabolic syndrome and metabolic syndrome components in young adults: A pooled analysis. Prev. Med. Rep..

[3] Boudreau D., Malone D.C., Raebel M., Fishman P., Nichols G., Feldstein A., Boscoe A., Ben-Joseph R., Magid D., Okamoto L. (2009). Health care utilization and costs by metabolic syndrome risk factors. Metab. Syndr. Relat. Disord..

[4] Gluckman P.D., Hanson M.A., Buklijas T., Low F.M., Beedle A.S. (2009). Epigenetic mechanisms that underpin metabolic and cardiovascular diseases. Nat. Rev. Endocrinol..

[5] Chen A.K., Roberts C.K., Barnard R.J. (2006). Effect of a short-term diet and exercise intervention on metabolic syndrome in overweight children. Metabolism.

[6] Church T. (2011). Exercise in obesity, metabolic syndrome, and diabetes. Prog. Cardiovasc. Dis..

[7] Moller D.E. (2001). New drug targets for type 2 diabetes and the metabolic syndrome. Nature.

[8] Sofowora A. (1996). Research on Medicinal Plants and Traditional Medicine in Africa. J. Altern. Complement. Med..

[9] McGaw L., Jäger A., Grace O., Fennel C., van Staden J. (2005). Medicinal plants. Ethics in Agriculture—An African Perspective.

[10] Kanwar P., Sharma N., Rekha A. (2006). Medicinal plants use in traditional healthcare systems prevalent in Western Himalayas. Ind. J. Trad. Know.

[11] Dillard C.J., German J.B. (2000). Phytochemicals: Nutraceuticals and human health. J. Sci. Food Agric..

[12] Rodriguez-Casado A. (2016). The health potential of fruits and vegetables phytochemicals: Notable examples. Crit. Rev. Food Sci. Nutr..

[13] Altemimi A., Lakhssassi N., Baharlouei A., Watson D., Lightfoot D. (2017). Phytochemicals: Extraction, isolation, and identification of bioactive compounds from plant extracts. Plants.

[14] Tomlinson T.R., Akerele O. (2015). Medicinal Plants: Their Role in Health and Biodiversity.

[15] Tabasum S., Khare S. (2016). Safety of medicinal plants: An important concern. Int. J. Pharm. Biol. Sci..

[16] Naghdi N. (2018). Folklore medicinal plants used in liver disease: A review. Int. J. Green Pharm. (IJGP).

[17] WHO World Health Organisation: Obesity. https://www.who.int/news-room/fact-sheets/detail/obesity-and-overweight.

[18] Caballero B. (2007). The global epidemic of obesity: An overview. Epidemiol. Rev..

[19] Peeters A., Barenddregt J.J., Willekens F., Mackenbach J.P., Mamum A.A., Bonneux L. (2003). Obesity in Adulthood and Its Consequences for Life Expectancy: Life-Table Analysis. Ann. Intern. Med..

[20] Nishida C., Mucavele P. (2005). Monitoring the rapidly emerging public health problem of overweight and obesity: The WHO Global Database on Body Mass Index. SCN News.

[21] Van Der Merwe M.T., Pepper M.S. (2006). Obesity in South Africa. Obes. Rev..

[22] SSA Statistics South Africa South Africa Demographic and Health Survey 2016: Key Indicator Report. https://www.statssa.gov.za/publications/Report%2003-00-09/Report%2003-00-092016.pdf.

[23] Kopelman P.G. (2000). Obesity as a medical problem. Nature.

[24] Goedecke J.H., Jenning C.L., Lambertc E.V. (2006). Chronic Diseases of Lifestyle in South Africa: 1995–2005. Medical Research Council–Technical Report.

[25] Field A.E., Coakley E.H., Must A., Spadano J.L., Laird N., Dietz W.H., Rimm E., Colditz G.A. (2001). Impact of overweight on the risk of developing common chronic diseases during a 10-year period. Arch. Intern. Med..

[26] Bray G. (2004). Medical consequences of obesity. J. Clin. Endocrinol. Metab..

[27] McTigue K.M., Harris R., Hemphill B., Lux L., Sutton S., Bunton A.J., Lohr K.N. (2003). Screening and interventions for obesity in adults: Summary of the evidence for the U.S. Preventive Services Task Force. Ann. Intern. Med..

[28] Howard A. (1981). The historical development, efficacy and safety of very-low -calorie diets. Int. J. Obes..

[29] Lowe M.R. (2003). Self-regulation of energy intake in the prevention and treatment of obesity: Is it feasible?. Obes. Res..

[30] Curioni C.C., Lourenco P.M. (2005). Long-term weight loss after diet and exercise: A systematic review. Int. J. Obes. Vol..

[31] Abdollahi M., Afshar-Imani B. (2003). A review on obesity and weight loss measures. Middle East Pharm..

[32] Yanovski S.Z., Yanovski J.A. (2014). Long-term drug treatment for obesity: A systematic and clinical review. J. Am. Med Assoc..

[33] IDF IDF Diabetes Atlas. **2017,** 8th Edition. https://www.idf.org/e-library/epidemiology-research/diabetes-atlas/134-idf-diabetes-atlas-8th-edition.html.

[34] Erasto P., Adebola P., Grierson D., Afolayan A. (2005). An ethnobotanical study of plants used for the treatment of diabetes in the Eastern Cape Province, South Africa. Afr. J. Biotechnol..

[35] Oyedemi S., Bradley G., Afolayan A. (2009). Ethnobotanical survey of medicinal plants used for the management of diabetes mellitus in the Nkonkobe municipality of South Africa. J. Med. Plants Res..

[36] Elghazaly N.A., Radwan E.H., Zaatout H.H., Elghazaly M.M., Allam N.E. (2019). Beneficial Effects of Fennel *(Foeniculum Vulgare)* in Treating Obesity in Rats. J. Obes. Manag..

[37] Pothurraju R., Sharma R.K., Chagalamarii J., Jangra S., Kavadi P.K. (2014). A systematic review of *Gymnema sylvestre* in obesity and diabetes management. J. Sci. Food Agric..

[38] Kanetkar P., Singhal R., Kamat M. (2007). *Gymnema sylvestre*: A Memoir. J. Clin. Biochem. Nutr..

[39] Van Heerden F.R. (2008). *Hoodia gordonii:* A natural appetite suppressant. J. Ethnopharmacol..

[40] Sibuyi N., Katerere D., Boboyi T., Madiehe A. (2007). Dietary supplementation with *Aloe ferox* extracts reverses obesity in rats. S. Afr. J. Bot..

[41] Shin E., Shim K.S., Kong H., Lee S., Shin S., Kwon J., Jo T.H., Park Y.I., Lee C.K., Kim K. (2011). Dietary Aloe improves insulin sensitivity via the suppression of obesity-induced inflammation in obese mice. Immune Netw..

[42] Misawa E., Tanaka M., Nabeshima K., Nomaguchi K., Yamada M., Toida T., Iwatsuki K. (2012). Administration of Dried *Aloe vera* Gel Powder Reduced Body Fat Mass in Diet-Induced Obesity (DIO) Rats. J. Nutr. Sci. Vitaminol..

[43] Zhang J., Kang M.J., Kim M.J., Kim M.E., Song J.H., Lee Y.M., Kim J.I. (2008). Pancreatic lipase inhibitory activity of *Taraxacum officinale* in vitro and *in vivo*. Nutr. Res. Pract..

[44] González-Castejón M., García-Carrasco B., Fernández-Dacosta R., Dávalos A., Rodriguez-Casado A. (2014). Reduction of Adipogenesis and Lipid Accumulation by *Taraxacum officinale* (Dandelion) Extracts in 3T3L1 Adipocytes: An in vitro Study. Phytother. Res..

[45] Afolayan A., Mbaebie B. (2010). Ethnobotanical study of medicinal plants used as anti-obesity remedies in Nkonkobe Municipality of South Africa. Pharmacogn. J..

[46] Clarke D.B. (2010). Glucosinolates, structures and analysis in food. Anal. Chem..

[47] Sansone R.A., Sansone L.A. (2014). Marijuana and body weight. Innov. Clin. Neurosci..

[48] Abifarin T.O., Afolayan A.J., Otunola G.A. (2019). Phytochemical and Antioxidant Activities of *Cucumis africanus* L.f.: A Wild Vegetable of South Africa. J. Evid.-Based Integr. Med..

[49] Vernarelli J.A., Lambert J.D. (2017). Flavonoid intake is inversely associated with obesity and C-reactive protein, a marker for inflammation, in US adults. Nutr. Diabetes.

[50] Unuofin J.O., Otunola G.A., Afolayan A.J. (2018). *In vitro* α-amylase, α-glucosidase, lipase inhibitory and cytotoxic activities of tuber extracts of *Kedrostis africana* (L.) Cogn. Heliyon.

[51] Ogawa S., Yazaki Y. (2018). Tannins from *Acacia mearnsi* De Wild. Mark: Tannin Determination and Biological Activities. Molecules.

[52] Ikarashi N., Toda T., Okaniwa T., Ito K., Ochiai W., Sugiyama K. (2011). Anti-obesity and anti-diabetic effects of acacia polyphenol in obese diabetic KKAy mice fed high-fat diet. Evid. Based Complementary Altern. Medcine.

[53] Bhat R.B., Moskovitz G. (2009). Herbal medicinal teas from South Africa. Fyton.

[54] Metwally F.M., Rashad H.M., Ahmed H.H., Mahmoud A.A., Raouf E.R.A., Abdalla A.M. (2017). Molecular mechanisms of the anti-obesity potential effect of *Moringa oleifera* in the experimental model. Asian Pac. J. Trop. Med..

[55] Bais S., Singh G.S., Sharma R. (2014). Antiobesity and Hypolipidemic Activity of *Moringa oleifera* Leaves against High Fat Diet-Induced Obesity in Rats. Adv. Biol..

[56] Vergara-Jimenez M., Almatrafi M.M., Fernandez M.L. (2017). Bioactive Components in *Moringa Oleifera* Leaves Protect against Chronic Disease. Antioxidants (BaselSwitz.).

[57] Choi E.-K., Cho Y.J., Yang H.J., Kim K.-S., Lee I.-S., Jang J.-C., Kim K.-H., Bang J.H., Kim Y., Kim S.H. (2015). Coix seed extract attenuates the high-fat induced mouse obesity via PPARγ and C/EBPα a downregulation. Mol. Cell. Toxicol..

[58] Lee S.H., Kim B., Oh M.J., Yoon J., Kim H.Y., Lee K.J., Lee J.D., Choi K.Y. (2011). *Persicaria hydropiper* (L.) spach and its flavonoid components, isoquercitrin and isorhamnetin, activate the Wnt/β-catenin pathway and inhibit adipocyte differentiation of 3T3-L1 cells. Phytother. Res..

[59] Odeyemi S.W., Afolayan A.J. (2018). Identification of Antidiabetic Compounds from Polyphenolic-rich Fractions of Bulbine abyssinica A. Rich Leaves. Pharmacogn. Res..

[60] Deutschländer M., Lall N., Van de Venter M., Hussein A.A. (2011). Hypoglycemic evaluation of a new triterpene and other compounds isolated from Euclea undulata Thunb. var. myrtina (Ebenaceae) root bark. J. Ethnopharmacol..

[61] Oyedemi S., Bradley G., Afolayan A. (2012). Antidiabetic activities of aqueous stem bark extract of *Strychnoshenningsii Gilg* in streptozotocin-nicotinamide type 2 diabetic rats. Iran. J. Pharm. Res. IJPR.

[62] Tiong S., Looi C., Hazni H., Arya A., Paydar M., Wong W., Cheah S.-C., Mustafa M., Awang K. (2013). Antidiabetic and antioxidant properties of alkaloids from Catharanthus roseus (L.) G. Don. Molecules.

[63] Oyedemi S., Koekemoer T., Bradley G., van de Venter M., Afolayan A. (2013). *In vitro* anti-hyperglycemia properties of the aqueous stem bark extract from *Strychnos henningsii* (Gilg). Int. J. Diabetes Dev. Ctries..

[64] Boaduo N.K.K., Katerere D., Eloff J.N., Naidoo V. (2014). Evaluation of six plant species used traditionally in the treatment and control of diabetes mellitus in South Africa using *in vitro* methods. Pharm. Biol..

[65] Akinrinde A., Koekemoer T., Van De Venter M., Bradley G. (2018). *In vitro* investigation of potential anti-diabetic activity of the corm extract of *Hypoxis argentea* Harv. Ex Baker. Acta Pharm..

[66] Sasidharan S., Sumathi V., Jegathambigai N.R., Latha L.Y. (2011). Antihyperglycaemic effects of ethanol extracts of *Carica papaya* and *Pandanus amaryfollius* leaf in streptozotocin-induced diabetic mice. Nat. Prod. Res..

[67] Semenya S., Potgieter M., Erasmus L. (2012). Ethnobotanical survey of medicinal plants used by Bapedi healers to treat diabetes mellitus in the Limpopo Province, South Africa. J. Ethnopharmacol..

[68] Thomson M., Al-Amin Z.M., Al-Qattan K.K., Shaban L.H., Ali M. (2007). Anti-diabetic and hypolipidaemic properties of garlic (*Allium sativum*) in streptozotocin-induced diabetic rats. Int. J. Diabetes Metab..

[69] Chadwick W.A., Roux S., van de Venter M., Louw J., Oelofsen W. (2007). Anti-diabetic effects of *Sutherlandia frutescens* in Wistar rats fed a diabetogenic diet. J. Ethnopharmacol..

[70] Ojewole J.A., Olayiwola G., Adewole S.O. (2006). Hypoglycaemic and hypotensive effects of Momordica charantia Linn (Cucurbitaceae) whole-plant aqueous extract in rats: Cardiovascular topics. Cardiovasc. J. S. Afr..

[71] Al-Shaqha W.M., Khan M., Salam N., Azzi A., Chaudhary A.A. (2015). Anti-diabetic potential of *Catharanthus roseus* Linn. and its effect on the glucose transport gene (GLUT-2 and GLUT-4) in streptozotocin induced diabetic wistar rats. BMC Complement. Altern. Med..

[72] Singh S.N., Vats P., Suri S., Shyam R., Kumria M., Ranganathan S., Sridharan K. (2001). Effect of an antidiabetic extract of *Catharanthus roseus* on enzymic activities in streptozotocin induced diabetic rats. J. Ethnopharmacol..

[73] Rasineni K., Bellamkonda R., Singareddy S.R., Desireddy S. (2010). Antihyperglycemic activity of Catharanthus roseus leaf powder in streptozotocin-induced diabetic rats. Pharmacogn. Res..

[74] Ibrahim M., Mehjabeen S., Narsu M.L. (2011). Pharmacological evaluation of *Catharanthus roseus*. Int. J. Pharm. Appl..

[75] Loots D.T., Pieters M., Shahidul Islam M., Botes L. (2011). Antidiabetic effects of Aloe ferox and Aloe greatheadii var. davyana leaf gel extracts in a low-dose streptozotocin diabetes rat model. S. Afr. J. Sci..

[76] Sunmonu T.O., Afolayan A.J. (2013). Evaluation of antidiabetic activity and associated toxicity of *Artemisia afra* aqueous extract in wistar rats. Evid.-Based Complement. Altern. Med..

[77] Van de Venter M., Roux S., Bungu L.C., Louw J., Crouch N.R., Grace O.M., Maharaj V., Pillay P., Sewnarian P., Bhagwandin N. (2008). Antidiabetic screening and scoring of 11 plants traditionally used in South Africa. J. Ethnopharmacol..

[78] Mellem J., Baijnath H., Odhav B. (2015). Antidiabetic potential of *Brachylaena discolor*. Afr. J. Tradit. Complement. Altern. Med..

[79] Van Huyssteen M., Milne P.J., Campbell E.E., van de Venter M. (2011). Antidiabetic and cytotoxicity screening of five medicinal plants used by traditional African health practitioners in the Nelson Mandela Metropole, South Africa. Afr. J. Tradit. Complement. Altern. Med..

[80] Frati A.C., Jiménez E., Ariza C.R. (1990). Hypoglycemic effect of Opuntia ficus indica in non insulin-dependent diabetes mellitus patients. Phytother. Res..

[81] Shin J.-E., Han M.-J., Lee I.-K., Moon Y.-I., Kim D.-H. (2003). Hypoglycemic activity of *Opuntia ficus-indica* var. sabotan on alloxan-or streptozotocin-induced diabetic mice. Korean. J. Pharm..

[82] Adeneye A., Olagunju J. (2009). Preliminary hypoglycemic and hypolipidemic activities of the aqueous seed extract of *Carica papaya* Linn in Wistar rats. Biol. Med.

[83] Omonkhua A.A., Onoagbe I.O., Ajileye A.F., Aladegboye L.O., Adetoboye A.R. (2013). Long term anti-diabetic, anti-hyperlipidaemic and anti-atherogenic effects of *Carica papaya* leaves in streptozotocin diabetic rats. Eur. J. Med. Plants.

[84] Ahmed I., Lakhani M., Gillett M., John A., Raza H. (2001). Hypotriglyceridemic and hypocholesterolemic effects of anti-diabetic *Momordica charantia* (karela) fruit extract in streptozotocin-induced diabetic rats. Diabetes Res. Clin. Pract..

[85] Ojewole J.A. (2006). Antinociceptive, anti-inflammatory and antidiabetic properties of *Hypoxis hemerocallidea* Fisch. & CA Mey.(Hypoxidaceae) corm [‘African Potato’] aqueous extract in mice and rats. J. Ethnopharmacol..

[86] Ojewole J. (2004). Analgesic, antiinflammatory and hypoglycemic effects of *Sutherlandia frutescens* R. BR.(variety Incana E. MEY.)(Fabaceae) shoot aqueous extract. Methods Find. Exp. Clin. Pharmacol..

[87] Zibula S.M., Ojewole J.A. (2000). Hypoglycaemic Effects of Hypoxis Hemerocallidea (Fisch. and CA Mey.) Corm’African Potato’Methanolic Extract in Rats. Med J. Islamic World Acad. Sci..

[88] Mahomed I., Ojewole J. (2003). Hypoglycemic effect of *Hypoxis hemerocallidea* corm (African potato) aqueous extract in rats. Methods Find. Exp. Clin. Pharmacol..

[89] Ojewole J. (2005). Antinociceptive, antiinflammatory and antidiabetic effects of Leonotis leonurus (L.) R. BR.(Lamiaceae) leaf aqueous extract in mice and rats. Methods Find. Exp. Clin. Pharmacol..

[90] Oyedemi S., Yakubu M., Afolayan A. (2011). Antidiabetic activities of aqueous leaves extract of *Leonotis leonurus* in streptozotocin induced diabetic rats. J. Med. Plants Res..

[91] Hu G., Qiao Q., Tuomilehto J., Balkau B., Borch-Johnsen K., Pyorala K. (2004). Prevalence of the metabolic syndrome and its relation to all-cause and cardiovascular mortality in nondiabetic European men and women. Arch. Intern. Med..

[92] Mayosi B.M., Flisher A.J., Lalloo U.G., Sitas F., Tollman S.M., Bradshaw D. (2009). The burden of non-communicable diseases in South Africa. Lancet.

[93] Michalsky M.P., Inge T.H., Jenkins T.M., Xie C., Courcoulas A., Helmrath M., Brandt M.L., Harmon C.M., Chen M., Dixon J.B. (2018). Cardiovascular risk factors after adolescent bariatric surgery. Pediatrics.

[94] Lawes C.M., Vander Hoorn S., Rodgers A. (2008). Global burden of blood-pressure-related disease, 2001. Lancet.

[95] Alberts M., Urdal P., Steyn K., Stensvold I., Tverdal A., Nel J.H., Steyn N.P. (2005). Prevalence of cardiovascular diseases and associated risk factors in a rural black population of South Africa. Eur. J. Cardiovasc. Prev. Rehabil..

[96] Tasos E., Huang A., Timimi L.J., Wych J., Mander A.P., Chowienczyk P.J., Wilkinson I.B., Mukhtar O. (2019). Randomised Controlled Trials of Anti-Hypertensive Therapy in Sub-Saharan Africa-A Systematic Review. Available SSRN 3327351.

[97] Rastogi S., Pandey M.M., Rawat A. (2016). Traditional herbs: A remedy for cardiovascular disorders. Phytomedicine.

[98] Rouhi-Boroujeni H., Heidarian E., Rouhi-Boroujeni H., Deris F., Rafieian-Kopaei M. (2017). Medicinal plants with multiple effects on cardiovascular diseases: A systematic review. Curr. Pharm. Des..

[99] Musabayane C.T., Kamadyaapa D.R., Gondwe M., Moodley K., Ojewole J.A. (2008). Cardiovascular effects of *Helichrysum ceres* S Moore [Asteraceae] ethanolic leaf extract in some experimental animal paradigms. Cardiovasc. J. Afr..

[100] Musabayane C. (2012). The effects of medicinal plants on renal function and blood pressure in diabetes mellitus. Cardiovasc. J. Afr..

[101] Kamadyaapa D.R., Gondwe M.M., Moodley K., Musabayane C.T., Ojewole J.A. (2009). Cardiovascular effects of *Ekebergia capensis* Sparrm (Meliaceae) ethanolic leaf extract in experimental animal paradigms. Cardiovasc. J. Afr..

[102] Bwititi P., Musabayane C., Nhachi C. (2000). Effects of Opuntia megacantha on blood glucose and kidney function in streptozotocin diabetic rats. J. Ethnopharmacol..

[103] Al-Qattan K., Thomson M., Ali M. (2008). Garlic (Allium sativum) and ginger (Zingiber officinale) attenuate structural nephropathy progression in streptozotocin-induced diabetic rats. e-SPENEur. E-J. Clin. Nutr. Metab..

[104] Braca A., Politi M., Sanogo R., Sanou H., Morelli I., Pizza C., De Tommasi N. (2003). Chemical composition and antioxidant activity of phenolic compounds from wild and cultivated *Sclerocarya birrea* (Anacardiaceae) leaves. J. Agric. Food Chem..

[105] Musabayane C., Gondwe M., Kamadyaapa D., Chuturgoon A., Ojewole J. (2007). Effects of *Ficus thonningii* (Blume)[Morarceae] stem-bark ethanolic extract on blood glucose, cardiovascular and kidney functions of rats, and on kidney cell lines of the proximal (LLC-PK1) and distal tubules (MDBK). Ren. Fail..

[106] Bennani-Kabchi N., Fdhil H., Cherrah Y., El F.B., Kehel L., Marquie G. (2000). Therapeutic effect of *Olea europea* var. oleaster leaves on carbohydrate and lipid metabolism in obese and prediabetic sand rats (Psammomys obesus). Ann. Pharm. Franc..

[107] Somova L., Shode F., Ramnanan P., Nadar A. (2003). Antihypertensive, antiatherosclerotic and antioxidant activity of triterpenoids isolated from *Olea europaea*, subspecies africana leaves. J. Ethnopharmacol..

[108] Cooper R., McFarlane-Anderson N., Bennett F.I., Wilks R., Puras A., Tewksbury D., Ward R., Forrester T. (1997). ACE angiotensinogen and obesity: a potential pathway leading to hypertension. J. Hum. Hypertens..

[109] Morgan T.O., Anderson A.I., MacInnis R.J. (2001). ACE inhibitors, beta-blockers, calcium blockers, and diuretics for the control of systolic hypertension. Am. J. Hypertens..

[110] Ramesar S., Baijnath H., Govender T., Mackraj I. (2008). Angiotensin I-converting enzyme inhibitor activity of nutritive plants in KwaZulu-Natal. J. Med. Food.

[111] Duncan A.C., Jäger A.K., van Staden J. (1999). Screening of Zulu medicinal plants for angiotensin converting enzyme (ACE) inhibitors. J. Ethnopharmacol..

[112] Lonardo A., Ballestri S., Marchesini G., Angulo P., Loria P. (2015). Nonalcoholic fatty liver disease: A precursor of the metabolic syndrome. Dig. Liver Dis..

[113] Lembede B.W. (2014). Effect of dietary Terminalia sericea aqueous leaf extracts on high-fructose diet fed growing Wistar rats. Master’s Thesis.

[114] Mazibuko S.E., Joubert E., Johnson R., Louw J., Opoku A.R., Muller C.J. (2015). Aspalathin improves glucose and lipid metabolism in 3T3-L1 adipocytes exposed to palmitate. Mol. Nutr. Food Res..

[115] Mazibuko-Mbeje S.E., Dludla P.V., Roux C., Johnson R., Ghoor S., Joubert E., Louw J., Opoku A.R., Muller C.J. (2019). Aspalathin-enriched green rooibos extract reduces hepatic insulin resistance by modulating PI3K/AKT and AMPK pathways. Int. J. Mol. Sci..

[116] Smith C., Krysman A. (2014). *Hoodia gordonii* extract targets both adipose and muscle tissue to achieve weight loss in rats. J. Ethnopharmacol..

[117] MacKenzie J., Koekemoer T.C., Roux S., van de Venter M., Dealtry G.B. (2012). Effect of *Sutherlandia frutescens* on the lipid metabolism in an insulin resistant rat model and 3T3-L1 adipocytes. Phytother. Res..

[118] Bhalla A., Chauhan U. (2015). Identification of antihyperlipidemic components in Aloe vera through reverse phase HPlC. J. Biol. Sci. Med..

[119] Muhammad N., Ibrahim K., Ndhlala A., Erlwanger K. (2019). *Moringa oleifera* Lam. prevents the development of high fructose diet-induced fatty liver. S. Afr. J. Bot..

[120] Joung H., Kim B., Park H., Lee K., Kim H.-H., Sim H.-C., Do H.-J., Hyun C.-K., Do M.-S. (2017). Fermented *Moringa oleifera* decreases hepatic adiposity and ameliorates glucose intolerance in high-fat diet-induced obese mice. J. Med. Food.

[121] Kang J.-W., Shin J.-K., Koh E.-J., Ryu H., Kim H.J., Lee S.-M. (2016). *Opuntia ficus-indica* seed attenuates hepatic steatosis and promotes M2 macrophage polarization in high-fat diet–fed mice. Nutr. Res..

[122] El-Hadary A.E., Ramadan Hassanien M.F. (2016). Hepatoprotective effect of cold-pressed *Syzygium aromaticum* oil against carbon tetrachloride (CCl4)-induced hepatotoxicity in rats. Pharm. Biol..

[123] Nyakudya T., Mukwevho E., Nkomozepi P., Erlwanger K. (2018). Neonatal intake of oleanolic acid attenuates the subsequent development of high fructose diet-induced non-alcoholic fatty liver disease in rats. J. Dev. Orig. Health Dis..

[124] Kumar Singh A., Cabral C., Kumar R., Ganguly R., Kumar Rana H., Gupta A., Rosaria Lauro M., Carbone C., Reis F., Pandey A.K. (2019). Beneficial effects of dietary polyphenols on gut microbiota and strategies to improve delivery efficiency. Nutrients.

[125] Carrera-Quintanar L., López Roa R.I., Quintero-Fabián S., Sánchez-Sánchez M.A., Vizmanos B., Ortuño-Sahagún D. (2018). Phytochemicals that influence gut microbiota as prophylactics and for the treatment of obesity and inflammatory diseases. Mediat. Inflamm..

[126] Li X., Chen Y., Lai Y., Yang Q., Hu H., Wang Y. (2015). Sustainable utilization of traditional chinese medicine resources: Systematic evaluation on different production modes. Evid.-Based Complement. Altern. Med..

